# Slip-resistant footwear reduces slips among National Health Service workers in England: a randomised controlled trial

**DOI:** 10.1136/oemed-2020-106914

**Published:** 2021-01-15

**Authors:** Sarah Cockayne, Caroline Fairhurst, Gillian Frost, Mark Liddle, Rachel Cunningham-Burley, Michael Zand, Catherine Hewitt, Heather M Iles-Smith, Lorraine Green, David J Torgerson

**Affiliations:** 1 Health Sciences, University of York, York, North Yorkshire, UK; 2 HSE Science and Research Centre, Health and Safety Executive, Buxton, Derbyshire, UK; 3 Science Divison - Economic and Social Analysis, Health and Safety Executive Bootle Headquarters, Bootle, Sefton, UK; 4 Shool of Health and Society, University of Salford, Salford, Greater Manchester, UK; 5 Chapel Allerton Hospital Leeds, NIHR Leeds Musculoskeletal Biomedical Research Unit, Leeds, UK

**Keywords:** health and safety, injury

## Abstract

**Objectives:**

Assess the effectiveness of 5* GRIP-rated slip-resistant footwear in preventing slips in the workplace compared to usual footwear (control group).

**Methods:**

A multicentre, randomised controlled trial; 4553 National Health Service (NHS) staff were randomised 1:1 to the intervention group (provided with 5* GRIP-rated slip-resistant footwear) or the control group. The primary outcome of incidence rate of self-reported slips in the workplace over 14 weeks was analysed using a mixed-effects negative binomial model. Secondary outcome measures included incidence rate of falls from a slip, falls not from a slip, proportion of participants reporting a slip, fall or fracture and time to first slip and fall.

**Results:**

6743 slips were reported: 2633 in the intervention group (mean 1.16 per participant, range 0 to 36) and 4110 in the control group (mean 1.80 per participant, range 0 to 83). There was a statistically significant reduction in slip rate in the intervention group relative to the control group (incidence rate ratio (IRR) 0.63, 95% CI 0.57 to 0.70, p<0.001). Statistically significant differences, in favour of the intervention group, were observed in falls from a slip (IRR 0.51, 95% CI 0.28 to 0.92, p=0.03), the proportion of participants who reported a slip (OR 0.58, 95% CI 0.50 to 0.66, p<0.001) or fall (OR 0.73, 95% CI 0.54 to 0.99, p=0.04) and time to first slip (HR 0.73, 95% CI 0.67 to 0.80, p<0.001).

**Conclusions:**

The offer and provision of 5* GRIP-rated footwear reduced slips in NHS staff in the workplace.

**Trial registration number:**

ISRCTN33051393.

**Key messages**

**What is already known about this subject?**

- There is some low-level evidence from previous studies to suggest that slip-resistant footwear can prevent slips, although it can be challenging to identify appropriate slip-resistant footwear due to the lack of robust testing and reliable information on which to base a decision.

**What are the new findings?**

- The Health and Safety Executive in Great Britain developed the GRIP-rating schemefor the assessment and classification of footwear slip resistance, and when 5* GRIP-rated footwear were tested in this trial, they were found to reduce the rate of slips by 37% and the rate of falls resulting from a slip by 49% among NHS employees.

**How might this impact on policy or clinical practice in the foreseeable future?**

- Slip-resistant footwear should be considered in areas where it is not possible to minimise or adequately control a potential slip risk.

## Introduction

Slips, trips and falls on the same level are a major cause of injury in the workplace. In Great Britain, they are a common cause of non-fatal injury to employees, accounting for around 30% of those reported to the Health and Safety Executive (HSE).^1^ An estimated 100 000 non-fatal workplace injuries occur each year due to a slip, trip or fall on the level,^2^ resulting in 867 000 annual lost working days (full day equivalent).^3^ The issue is compounded by an ageing workforce.^4^ Those at older ages are more likely to fall and sustain more serious injuries when they do fall.^5 6^

Several factors can contribute to slips in the workplace including: the type of floor surface, whether it is contaminated, how it is cleaned and the type of footwear worn. In situations where it is impossible to prevent the floor becoming slippery, slip-resistant footwear should be considered to minimise the risk of slips. There is evidence to suggest that slip-resistant footwear is effective. A longitudinal study evaluating a slip, trip and fall prevention programme in US hospital workers,^7^ which included slip-resistant shoes among other strategies, reported a 58% reduction in workers’ compensation claims. A cluster randomised controlled trial (RCT) that recruited food service workers from 226 school districts in the USA, showed the probability of experiencing a slip injury was reduced by 67% in those allocated to receive appropriately specified slip-resistant shoes.^8^

Selecting appropriate slip-resistant footwear can be challenging as there is a lack of robust testing and reliable information on which to base a decision. The HSE in Great Britain developed the GRIP-rating scheme to measure and grade the slip resistance of footwear.^9^ This scheme goes beyond the minimum level required under the British and European standards;^10^ it evaluates footwear in simulated, ‘lifelike’ conditions, which provides a more accurate indication of the slip-resistant properties of the footwear. The Stopping Slips among Healthcare Workers (SSHeW) trial evaluated the effectiveness of 5* GRIP-rated slip-resistant footwear (the highest possible rating) to reduce slips in National Health Service (NHS) employees working in general, clinical or catering areas. This article reports the effectiveness results; economic and qualitative evaluations were undertaken and will be reported elsewhere.

## Methods

### Study design

Between March 2017 and January 2019, we enrolled participants in this two-arm, multicentre RCT at seven NHS trusts in England. Details of the trial design and implementation are provided in the published protocol.^11^

### Participants

NHS employees were provided with trial information, which included a consent form, and a baseline questionnaire to collect eligibility and demographic data. Participants were eligible for the trial if they: worked in a clinical, general or catering area; were aged 18 years and over; worked at least 22.5 hours a week; had a mobile phone; and were willing to receive/send text messages for data collection. We excluded them if they were: required to wear protective footwear by their employer; predominantly office or theatre-based; agency staff or staff with less than 6 months remaining on their employment contract. All participants provided written informed consent.

### Changes to study design

To aid recruitment, after input from the TSC/DMEC (TrialSteering and Data Monitoring and Ethics committee) and with the funder’s agreement, the eligibility criterion regarding working hours was revised in April 2018. Initially participants were required to work, on average, at least 80% working time equivalent (WTE), 30 hours per week. This was reduced to 60% WTE (22.5 hours per week). Participating sites had saturated the eligible workforce and found that the number of hours worked was the predominant factor limiting others from taking part. The required number of working hours was dropped to no lower than 22.5 so participants were working a sufficient amount of time to (a) ensure a sufficient work-related slip rate and (b) retain the ability to detect a reduction in this slip rate. It was felt that if the working hours had been reduced further, then it would not have been possible to do this.

### Randomisation and masking

Participants were inducted into the trial with a welcome text message followed by a pre-randomisation ‘run-in’ period of data collection in the form of weekly text messages, for up to 6 weeks, requesting data on slips at work in the previous week. The purpose of the ‘run-in’ period was twofold: to provide a baseline slip rate; and to assess for engagement with the text message data collection process. By only randomising participants who provided slip data for at least 2 weeks, we hoped to maximise response to the post-randomisation outcome texts. Participants were randomised 1:1 to either be offered and provided with a pair of 5* GRIP-rated slip-resistant shoes or asked to wear their usual work shoes. Randomisation was through the University of York, York Trials Unit (YTU) secure, remote, web-based randomisation service. The allocation sequence was generated by an independent data systems manager at YTU, who was not involved in the recruitment of participants. Sites informed YTU when they had capacity to order and deliver footwear. Block randomisation, stratified by trust, was used. The block size varied and was equal to the number of participants to be randomised in each batch, which was determined by site capacity (range 2 to 335, median 14). Participants and members of the study team including the statistician, health economist and those involved in the administration of the study were not masked to treatment allocation.

### Intervention

**Figure 1:**
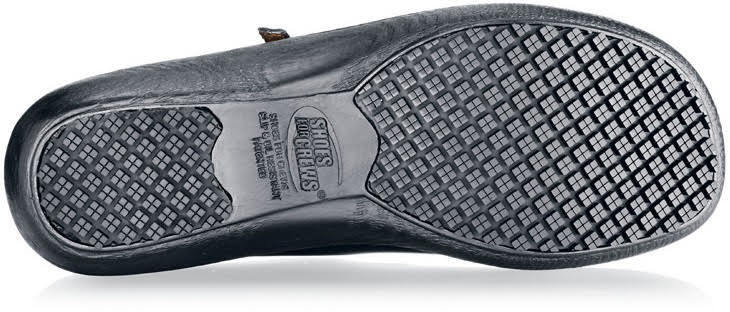
Example of sole of intervention footwear.

Intervention arm participants were offered and provided with a pair of 5* GRIP-rated slip-resistant shoes^12^ to wear during the 14-week trial period. In the GRIP-rating scheme, shoe manufacturers pay to have their footwear tested. Depending on their performance, the footwear is assigned a rating from one to five stars, with five indicating the best performing. The footwear for this trial was provided by a single manufacturer (Shoes For Crews Ltd), who had no input into the design, conduct or analysis of the study. This company was selected as it was able to supply large numbers of 5* GRIP-rated shoes in various styles over a wide geographical area. The cost of the footwear was covered by the NHS trust or trust’s charities. The shoes had a rubber sole with an intricate tread pattern (figure 1). The tread provided a large surface contact area while also being able to disperse surface contamination. However, when walking on clean and dry surfaces, the footwear was expected to behave like any other footwear.

### Control

Control arm participants continued to wear their usual work shoes for the 14-week trial period. Trusts’ footwear policy stated footwear should be sensible, low-heeled and not open-toed. Only four of the trusts stipulated that non-slip footwear should be worn, but none stated how this should be assessed. After the trial, control participants were offered a free pair of the 5* GRIP-rated slip-resistant shoes.

### Follow-up

Participants were sent weekly text messages for 14 weeks to collect slip outcome data and a 14-week post-randomisation follow-up postal questionnaire. On reporting their first slip, participants were sent a slip data collection questionnaire to ask whether they were wearing the trial shoes when they slipped, location of slip and any resulting injuries. If an injury was reported this was followed up with monthly data collection until the injury resolved, the participant no longer wished to be contacted or the trial ended. Participants in the intervention group were also sent text messages at 6, 10 and 14 weeks post-randomisation to collect data on how often they wore the trial shoes.

### Outcomes

The primary outcome was the incidence rate of self-reported slips, not necessarily resulting in a fall or injury, in the workplace over the 14 weeks from randomisation, as reported via weekly text messages (or the 14-week questionnaire where no text messages were received). A slip was defined as ‘a loss of traction of your foot on the floor surface, which may or may not result in a fall’. A fall was defined as ‘an unexpected event in which you come to rest on the ground, floor or lower level’. Secondary outcomes included the incidence rate of falls both resulting from a slip and not resulting from a slip in the workplace over 14 weeks, the proportion of participants who reported a slip, fall or fracture over 14 weeks and time to first fall and first slip. Other data collected included: issues with wearing footwear and adverse events, which were collected via the 14-week follow-up, slip data collection and injury questionnaires or responses to text messages; and compliance with wearing the trial shoes, which was collected via the 14-week questionnaire and text messages sent at 6, 10 and 14 weeks.

### Sample size

There were limited published data on which to base a sample size calculation for this trial. A prospective cohort study^13^ found that 49 of 422 (11.6%) workers in a restaurant setting in the USA reported at least one ‘major’ (ie, resulting in a fall and/or injury) slip over a 12-week follow-up period. It was therefore expected that the proportion of workers that experienced any type of slip would be higher than this, though the exact figure was not reported. For this sample size calculation, a conservative estimate of a proportion of 10% of the control group that would experience at least one slip over a 14-week follow-up period was assumed. Randomising 4400 participants 1:1 (ie, 2200 per group) would provide 90% power to show a 30% relative reduction in the proportion of participants who report at least one slip over 14-weeks (3 percentage point absolute reduction from 10% to 7%) allowing for 20% attrition. This sample size would also give 80% power to see an absolute reduction in the risk of falls from 5.5% to 3.5% allowing for 20% attrition. Although the sample size calculation was based on detecting a difference in proportions, the primary outcome was the incidence rate of slips over the 14 weeks and so negative binomial regression was used to compare this outcome between the two groups. Since this analysis used more information than a simple binary outcome, it was considered to be adequately powered.

### Statistical analysis

Statistical analyses were conducted in Stata V.15 (StataCorp, 4905 Lakeway Drive, College Station, Texas 77845, USA), following the principles of intention-to-treat analysing participants in the groups to which they were originally assigned regardless of compliance (or not) with allocation, using two-sided significance of 0.05. The follow-up period for all participants commenced on the date of randomisation and lasted a maximum of 14 weeks; therefore, for intervention participants, this often included some weeks at the start when they had not yet received their shoes. For the primary analysis of the incidence rate of slips, a mixed-effects negative binomial model was used adjusting for gender, job role and baseline weekly slip rate as fixed effects and site as a random effect. The model took account of the number of weeks for which slip data were provided and the total hours worked during those weeks. The person-working-hours were calculated by multiplying the number of weeks for which the participant provided slip data by the average number of hours they told us they tended to work in a week. We assumed participants were working in the weeks for which they provided data. The primary analysis was undertaken by CF and checked by GF. Complier average causal effect (CACE) analyses were undertaken for the primary outcome to account for the number of weeks participants had their shoes and the amount of time they reported wearing them.

The incidence rate of falls (both resulting and not resulting from a slip) over 14 weeks was analysed in the same way as the primary outcome. Mixed-effects logistic regression adjusted as for the primary analysis was used to compare, between the two groups, the proportions of participants who: (1) slipped at least once over 14 weeks; and (2) fell at least once over 14 weeks. The time from randomisation to first slip was analysed by Cox proportional hazards regression. Proportion of participants reporting a fracture and time to first fall were summarised descriptively but not formally analysed due to the small number of events.

### Public involvement

The SSHeW study was informed by the involvement of NHS staff (aged 20 to 71 years) from diverse roles including nurses, catering, housekeeping and doctors. They provided feedback about: rationale for the trial; shoe styles; use of text messages to collect data; use of a slip diary; the length of the follow-up; footwear buying habits; and testing slip-resistance of staff’s usual shoes. An NHS employee was a member of the TSC/DMEC.

## Results

**Figure 2:**
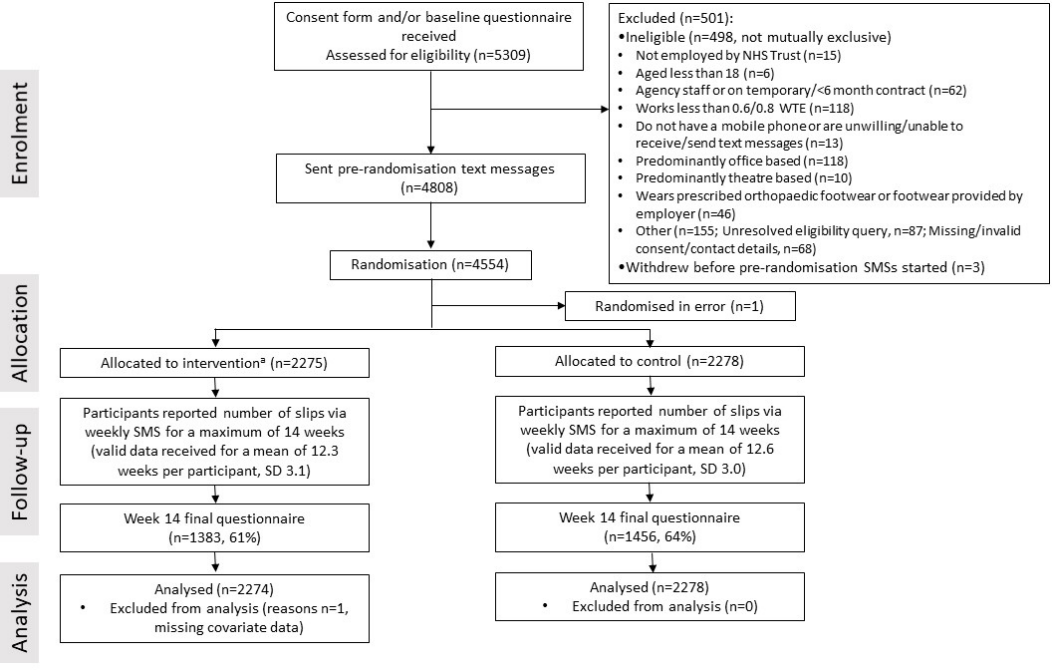
Flow of participants through trial. ^a^n=1930, 85% received trial shoes within 14-week trial follow-up. NHS, National Health Service; SMSs, short messaging services; WTE, working time equivalent.

Participants were recruited between March 2017 and November 2018 and randomised between June 2017 and January 2019. Trial invitation packs were sent to 8524 NHS staff, with 5309 (62.3%) returning a consent form and baseline questionnaire indicating they wished to take part. A total of 4808 were eligible and were sent up to six pre-randomisation, weekly texts. Of these, 4554 participants provided valid slip data to at least two of the texts and were randomised into the trial (intervention group, n=2276; control group, n=2278). One participant was immediately withdrawn as they were found to be ineligible. Figure 2 shows the participant flow during the trial and reports the reasons why participants were ineligible for the study.

### Participant characteristics

Baseline characteristics were well balanced across groups (table 1, and see online supplemental 1). Most participants were women (n=3869, 85.0%) and the average age was 42.7 years (range 18 to 74). Participants worked a median of 37.5 hours a week, and the majority were qualified nurses/midwives (n=1937, 42.5%). Just over one-third reported at baseline that they had experienced a slip at work in the previous 12 months (median of two slips), of which 11% had suffered an injury as a result of one of these slips.

10.1136/oemed-2020-106914.supp1 Supplementary data

**Table 1:** Characteristics of the participants

Characteristic	Intervention (n=2275)	Control (n=2278)	Total (n=4553)
Age, years			
Mean (SD)	42.7 (11.5)	42.7 (11.3)	42.7 (11.4)
Median (min, max)	44.0 (19.0 to 74.0)	44.0 (18.0 to 71.0)	44.0 (18.0 to 74.0)
Gender (Female), n (%)	1948 (85.6)	1921 (84.3)	3869 (85.0)
Average number of hours worked per week			
Mean (SD)	35.8 (4.3)	35.8 (4.5)	35.8 (4.4)
Median (min, max)	37.5 (22.5 to 63.0)	37.5 (15.0 to 62.0)	37.5 (15.0 to 63.0)
Job type, n (%)			
Qualified nurse/midwife	952 (41.8)	985 (43.2)	1937 (42.5)
Support worker	286 (12.6)	276 (12.1)	562 (12.3)
Healthcare assistant	272 (12.0)	246 (10.8)	518 (11.4)
Other qualified staff/healthcare professional	150 (6.6)	163 (7.2)	313 (6.9)
Domestic services	115 (5.1)	110 (4.8)	225 (4.9)
Administrator/receptionist/secretarial	78 (3.4)	100 (4.4)	178 (3.9)
Occupational therapist	65 (2.9)	65 (2.9)	130 (2.9)
Imaging	64 (2.8)	53 (2.3)	117 (2.6)
Physiotherapist	68 (3.0)	36 (1.6)	104 (2.3)
Pharmacist/pharmacy technician	50 (2.2)	53 (2.3)	103 (2.3)
Ward clerk	44 (1.9)	49 (2.2)	93 (2.0)
Doctor/consultant	38 (1.7)	42 (1.8)	80 (1.8)
Catering	29 (1.3)	38 (1.7)	67 (1.5)
Other	51 (2.2)	45 (2.0)	96 (2.1)
Areas worked in, n (%)*			
Ward	1261 (55.4)	1199 (52.6)	2460 (54.0)
Clinical room/area	722 (31.7)	755 (33.1)	1477 (32.4)
Community	260 (11.4)	268 (11.8)	528 (11.6)
Indoor hospital grounds/corridors	160 (7.0)	183 (8.0)	343 (7.5)
Office	91 (4.0)	113 (5.0)	204 (4.5)
Food preparation/serving area	77 (3.4)	87 (3.8)	164 (3.6)
Other	80 (3.5)	118 (5.2)	198 (4.3)
Had slip at work in the 12 months prior to enrolling in the study, n (%)	850 (37.4)	885 (38.8)	1735 (38.1)
If yes, how many? Median (min, max)	2 (1 to 400)	2 (1 to 300)	2 (1 to 400)
Suffered injury from any of these slips, n (%)	96 (11.3)	89 (10.1)	185 (10.7)

*not mutually exclusive.

%, percentage; max, maximum; min, minimum; n, number; SD, standard deviation.

### Primary outcome

At least 1 weeks’ worth of post-randomisation SMS (short messaging service) slip data were available from 4494 randomised participants (98.7%; intervention group, n=2254, 99.1%; control, n=2240, 98.3%) (see online supplemental 2). A further 11 (intervention group, n=4; control group, n=7) did not respond to any text messages, but provided data for number of slips experienced on their 14-week questionnaire, so were included in the primary model. The primary model also included participants who did not provide any post-randomisation slip data, by considering that they reported zero slips over a minimal exposure time of 0.1 hours. In total, the intervention group reported 2633 slips over 28 002 person-weeks, which equates to approximately 1 001 959 person-working-hours (mean per randomised participant 1.16, SD 2.9, median 0, range 0 to 36), and the control group reported 4110 slips over 28 595 person-weeks, or 1 025 180 person-working-hours (mean 1.80, SD 4.6, median 0, range 0 to 83). In total, 1824/4505 (40.5%) participants reported at least one slip. Information about a participant’s first slip was received for 1159 (63.5%) (intervention group, 61.9%; control group, 65.0%) (see online supplemental 3). Nearly all occurred on the ward (96.9%) and over three-quarters on a smooth surface. Only 3.0% of slips lead to a fall and most did not result in an injury (86.4%). There were no reported fractures from the first reported slip, though one control participant later reported a broken ankle due to a subsequent slip.

The incidence rates of slips per person-working-week were approximately 0.10 (95% CI 0.09 to 0.11) in the intervention group and 0.15 (95% CI 0.14 to 0.17) in the control group. There was a 37% reduction in the slip rate in the intervention group relative to the control group (incidence rate ratio (IRR) 0.63, 95% CI 0.57 to 0.70, p<0.001) (table 2). Of the 2275 intervention participants, 1523 (66.9%) received a pair of trial shoes within 7 weeks of randomisation, and 1930 (84.8%) within 14 weeks (median 27 days post-randomisation). Some participants either did not receive or were delayed in receiving their footwear due to issues with ordering, distributing or collecting footwear. Of the 28 002 person-weeks of slip data provided by intervention participants, we estimate two-thirds (18 696, 66.8%) of these were after they had received their shoes. Compliance data indicated that, once they had received their trial footwear, 50% of intervention participants wore the shoes all of the time while at work. A similar treatment estimate was observed when CACE analysis explored the impact of receiving shoes within seven (IRR 0.65, 95% CI 0.58 to 0.73, p<0.001) and 14 weeks (IRR 0.65, 95% CI 0.59 to 0.73, p<0.001) (table 2). A compliance ‘score’ was also computed, which incorporated when shoes were received and the amount of time they were worn, where a higher score indicated greater compliance. The intervention group scored, on average, 5.2/12 (SD 4.4); the control group all scored 0. The CACE analysis indicated that for every unit increase in compliance score, the rate of slipping was reduced by approximately 5% (IRR 0.95, 95% CI 0.94 to 0.97, p<0.001).

**Table 2:** Summary of analysis results

Outcome and analysis	Intervention (n=2275)	Control (n=2278)	Adjusted treatment effect estimate (95% CI)	P value
Primary analysis*				
Total number of slips	2633	4110		
Mean (SD)	1.16 (2.9)	1.80 (4.6)	IRR 0.63 (0.57 to 0.70)	<0.001
CACE sensitivity analyses*				
Receiving shoes within 7 weeks, n (%)	1523 (66.9)	0 (0.0)	IRR 0.65 (0.58 to 0.73)	<0.001
Receiving shoes within 14 weeks, n (%)	1930 (84.8)	0 (0.0)	IRR 0.65 (0.59 to 0.73)	<0.001
Continuous score (out of a possible 12), mean (SD)	5.2 (4.4)	0.0 (0.0)	IRR 0.95 (0.94 to 0.97)	<0.001
Secondary analyses*				
1+slip, n (%)	804 (35.6)	1020 (45.4)	OR 0.58 (0.50 to 0.66)	<0.001
1+fall†, n (%)	80 (3.5)	107 (4.7)	OR 0.73 (0.54 to 0.99)	0.04
Number of falls from a slip, mean (SD)	0.02 (0.22)	0.03 (0.23)	IRR 0.51 (0.28 to 0.92)	0.03
Number of falls *not* from a slip, mean (SD)	0.13 (0.9)	0.33 (2.1)	IRR 0.82 (0.50 to 1.34)	0.44
Time to first slip, 0.33 percentile days (95% CI)	46 (34 to 60)	27 (25 to 32)	HR 0.73 (0.67 to 0.80)	<0.001

*over 14 weeks.

†including those resulting from a slip and those not resulting from a slip.

%, percentage; CACE, complier average causal effect; CI, confidence interval; HR, hazard ratio; IRR, incidence rate ratio; n, number; OR, odds ratio; SD, standard deviation.

### Secondary outcomes

Statistically significant findings, favouring the slip-resistant footwear, were seen in the following secondary outcomes: falls resulting from a slip in the workplace (IRR 0.51, 95% CI 0.28 to 0.92, p=0.03), the proportion of participants who reported a slip (OR 0.58, 95% CI 0.50 to 0.66, p<0.001) or fall (OR 0.73, 95% CI 0.54 to 0.99, p=0.04) and time to first slip (HR 0.73, 95% CI 0.67 to 0.80, p<0.001) (table 2). There was no evidence of a difference in falls not resulting from a slip (IRR 0.82, 95% CI 0.50 to 1.34, p=0.44). First falls were reported a median of 34 days after randomisation in the intervention group and 41 days in the control group. There were no related serious adverse events reported.

## Discussion

### Principal findings

This randomised controlled trial showed that 5* GRIP-rated slip-resistant footwear reduced the rate of slips among NHS workers by 37%. Reductions were also seen in the proportion of participants who had a slip or fall.

### Comparison to other studies

As far as we are aware there is only one previous RCT evaluating slip-resistant footwear.^8^ This differed in its design, as it was cluster randomised, and population as it involved food service workers in US schools (only 1.5% of SSHeW participants were food service workers). The study also differed in its primary focus, which was to prevent workers’ compensation injury claims; therefore, it assessed slipping injuries rather than slips in general. A 67% reduction in the likelihood of reporting a slipping injury at follow-up relative to baseline was observed in the intervention arm, and this decrease was statistically significantly higher than was seen in the control group. The SSHeW results are broadly consistent with non-trial evidence supporting the benefits of slip-resistant footwear including: a before and after study of Danish fishermen^14^ observed an 80% reduction in slips, trips and falls associated with wearing new boots with ‘anti-slipping soles’; a 58% reduction in compensation claims when a slip, trip and fall prevention programme was introduced in a US hospital;^8^ and a 54% reduction in self-reported slipping in a cohort study involving fast food restaurant workers.^13^

### Strengths and weaknesses of the study

The strengths of this study include its robust methodology, large sample size and high engagement. Almost every participant (98.7%) provided at least one valid response to the primary outcome text messages. The short (14-week) follow-up, use of a run-in period and weekly data collection may have contributed to the low post-randomisation attrition rate and minimised recall bias.

There are certain limitations to this study. Outcome data were participant self-reported and it wasn’t possible to blind participants to group allocation, which may have led to under-reporting or over-reporting of slips. Approximately 15% of intervention participants failed to receive their shoes within the 14-week timeframe. These were considered non-compliant for the entire period, but were analysed as part of the intervention group under the principles of intention to treat, which may have diluted the observed treatment effect.

### Meaning of the study

The preference would always be to try to minimise or adequately control a potential slip risk for individuals by eliminating, for example, floor surface contamination; however, this may not always be possible. In this situation, employers or staff may consider the use of slip-resistant footwear. The SSHeW study has shown clear evidence that the 5* GRIP-rated footwear used in this study can reduce slips, and falls resulting from slips, in the NHS workplace. There is, therefore, a role for appropriately-specified footwear in reducing slips in the workplace.

### Unanswered questions and future directions

While the participants represented a diverse working group, our findings may not generalise to all workplaces. The trial should be replicated in other high-risk environments, such as manufacturing and food processing facilities or in settings that have different surfaces, such as in the construction industry.

## Data Availability

Data are available upon reasonable request. All data requests should be made to the corresponding author and will be considered on a case-by-case basis by the Trial Management Group. All data requests will be managed in accordance with York Trials Unit, University of York, processes and procedures.
