# Supplementary material for: Slip-resistant footwear reduces slips among National Health Service workers in England: a randomised controlled trial
Source: Occup Environ Med. 2021 Jan 15;78(7):472–8. doi: 10.1136/oemed-2020-106914 (PMC8223622; doi:10.1136/oemed-2020-106914)
Supplement: Supplementary data [file oemed-2020-106914supp001.pdf]

Slip-resistant footwear reduces slips among National Health Service workers in England: A randomised controlled trial

Supplementary 1 Characteristics of the participants

Supplementary 2 Response rates to post-randomisation messages

Supplementary 3 Data collected on the first slip reported by participants

SSHeW supplementary material v4

Supplementary 1 Characteristics of the participants

Characteristic	Intervention (n=2275)	Control (n=2278)	Total (n=4553)
**Time spent on feet at work, n (%)**			
Most of the time	1843 (81.0)	1829 (80.3)	3672 (80.7)
Some of the time	385 (16.9)	400 (17.6)	785 (17.2)
A little of the time	11 (0.5)	18 (0.8)	29 (0.6)
Missing	36 (1.6)	31 (1.4)	67 (1.5)
**Required to work in the community, n (%)**			
Yes	552 (24.3)	554 (24.3)	1106 (24.3)
No	1688 (74.2)	1689 (74.1)	3377 (74.2)
Missing	35 (1.5)	35 (1.5)	70 (1.5)
**Have you had a fall at work in the past 12 months?, n (%)**			
Yes	188 (8.3)	192 (8.4)	380 (8.3)
No	2039 (89.6)	2040 (89.6)	4079 (89.6)
Don't know	29 (1.3)	31 (1.4)	60 (1.3)
Missing	19 (0.8)	15 (0.7)	34 (0.7)
**If yes, how many?**			
Median (min, max)	1 (1, 52)	1 (1, 20)	1 (1, 52)
**Have you suffered injury from any of these falls?, n (%)**	69 (36.9)	74 (39.5)	144 (38.2)

max, maximum; min, minimum; n, number; %, percentage

Supplementary 2 Response rates to post-randomisation messages

Week post-randomization	Intervention (n=2275)	Control (n=2278)	Total (n=4553)
	N received / N sent (%)	N received / N sent (%)	N received / N sent (%)
1	2109/2275 (92.7)	2112/2278 (92.7)	4221/4553 (92.7)
2	2117/2272 (93.2)	2101/2278 (92.2)	4218/4550 (92.7)
3	2079/2270 (91.6)	2094/2278 (91.9)	4173/4548 (91.8)
4	2052/2269 (90.4)	2075/2276 (91.2)	4127/4545 (90.8)
5	2063/2267 (91.0)	2068/2275 (90.9)	4131/4542 (91.0)
6	2033/2265 (89.8)	2047/2272 (90.1)	4080/4537 (89.9)
7	1967/2265 (86.8)	2055/2271 (90.5)	4022/4536 (88.7)
8	1963/2261 (86.8)	2028/2271 (89.3)	3991/4532 (88.1)
9	1962/2261 (86.8)	2034/2271 (89.6)	3996/4532 (88.2)
10	1936/2259 (85.7)	2012/2271 (88.6)	3948/4530 (87.2)
11	1948/2259 (86.2)	1996/2271 (87.9)	3944/4530 (87.1)
12	1926/2256 (85.4)	1988/2271 (87.5)	3914/4527 (86.5)
13	1921/2255 (85.2)	1996/2271 (87.9)	3917/4526 (86.5)
14	1924/2253 (85.4)	1985/2269 (87.5)	3909/4522 (86.4)
Response to at least one text message	2254/2275 (99.1%)	2240/2278 (98.3%)	4494/4553 (98.7%)
Response to all 14 text messages	1289/2275 (56.7%)	1400/2278 (61.5%)	2689/4553 (59.1%)

N, number, %, percentage

SSHeW supplementary material v4

Supplementary 3 Data collected on the first slip reported by participants

Characteristic	Intervention (n=497)	Control (n=662)	Total (n=1159)
**Hours worked in week of first slip**			
Mean (SD)	35.7 (7.8)	35.6 (7.0)	35.6 (7.4)
Median (min, max)	37.5 (3.5, 75.0)	37.5 (5.0, 75.0)	37.5 (3.5, 75.0)
**Type of slip, n (%)**			
Slip without falling (however minor)	437 (87.9)	596 (90.0)	1033 (89.1)
Slip and fall	12 (2.4)	23 (3.5)	35 (3.0)
Missing	48 (9.7)	43 (6.5)	91 (7.9)
**Wearing trial shoes when slipped, n (%)**			
Yes	82 (16.5)	-	-
No	363 (73.0)	-	-
Don’t know	3 (0.6)	-	-
Missing	49 (9.9)	-	-
**Location of slip, n (%)**			
On a ward or other clinical area in a hospital	479 (96.4)	644 (97.3)	1123 (96.9)
In a non-clinical area in a hospital e.g. office, corridor	5 (1.0)	3 (0.5)	8 (0.7)
In a catering area where food is prepared or served	1 (0.2)	0 (0.0)	1 (0.1)
Outside	4 (0.8)	6 (0.9)	10 (0.9)
Inside a patient's home	1 (0.2)	0 (0.0)	1 (0.1)
Other	7 (1.4)	9 (1.4)	16 (1.4)
Missing	0 (0.0)	0 (0.0)	0 (0.0)
**Slipped on…, n (%)**			
Smooth surface	341 (68.6)	532 (80.4)	873 (75.3)
Textured surface	92 (18.5)	77 (11.6)	169 (14.6)
Missing	64 (12.9)	53 (8.0)	117 (10.1)
**Injuries suffered, n (%)[a]**			
None	419 (84.3)	582 (87.9)	1001 (86.4)
Superficial wound	12 (2.4)	17 (2.6)	29 (2.5)
Broken bone	0 (0.0)	0 (0.0)	0 (0.0)
Pulled muscle/sprained ligament	10 (2.0)	12 (1.8)	22 (1.9)
Other	5 (1.0)	3 (0.5)	8 (0.7)
Missing	52 (10.5)	49 (7.4)	101 (8.7)

[a] not mutually exclusive

SD, standard deviation; max, maximum; min, minimum; n, number; %, percentage

SSHeW supplementary material v4
